# Suicidal overdose of dolutegravir: A case report

**DOI:** 10.4102/sajhivmed.v19i1.799

**Published:** 2018-06-18

**Authors:** Rahul Daimari, Lawrence Kwape, Anthony A. Oyekunle

**Affiliations:** 1 Department of Internal Medicine, University of Botswana, Botswana; 2 Princess Marina Hospital, Botswana

## Abstract

Dolutegravir (DTG) is the most recently introduced integrase inhibitor for the treatment of HIV infection and is preferred for its superior tolerability and efficacy in both new and pre-treated patients, and infrequent drug interactions. Since January 2017, Botswana has adopted a ‘treat-all’ approach with a DTG-based antiretroviral (ARV) regimen as first-line treatment. We report a 29-year-old man with clinical stage 1 HIV infection who had been started on DTG, tenofovir and emtricitabine eight months prior, and who was admitted following a suicidal overdose of 1500 mg of DTG. He reported only minor symptoms including vomiting, epigastric pain and dizziness; which promptly resolved following supportive treatment. On admission, full blood count, liver function tests and electrocardiography were unremarkable. However, there was a non-progressive increase in serum creatinine. After a month off ARVs, he was successfully restarted on antiretroviral therapy without any serious adverse effect.

## Background

Integrase strand transfer inhibitors (InSTIs) are the newest class of antiretroviral (ARV) drugs approved for HIV treatment. Combination therapy based on InSTI is one of the alternative first-line ARV drugs recommended by the World Health Organization (WHO).^1^ Dolutegravir (DTG) is the most recent InSTI and is preferred for its superior tolerability and efficacy in both new and treatment-experienced patients and infrequent drug-drug interactions.^2,3^ Since June 2016, Botswana has adopted the ‘universal test and treat’ approach for HIV-infected persons, with a DTG-based regimen.^4^

## Ethical consideration

For this case report, the authors obtained ethical approval from the Research and Ethics Committee (REC) of Princess Marina Hospital, Gaborone, and written informed consent from the patient. Ethics approval number is PMH 5/79(1-4-2018).

## Case

We report a 29-year-old HIV-positive male truck driver, who was diagnosed with WHO clinical stage 1 HIV infection in May 2016, with a CD4+ count of 317 cells/µL, and was started on a triple regimen comprising DTG 50 mg and tenofovir (TDF) 300 mg and emtricitabine (FTC) 200 mg in September 2016. The TDF/FTC were in a fixed dose combination, and DTG was provided as a separate pill. He achieved virologic suppression, with a viral load < 400 copies/mL in December 2016. However, after an argument with his girlfriend, he ingested 30 tablets of DTG (1500 mg in total) with suicidal intention. He denied taking the other antiretroviral (ARV) drugs (TDF/FTC). He arrived at our hospital about 2 h after the incident and complained of epigastric pain, dizziness and vomiting. There was no prior history of depression or psychiatric illness. He drank alcohol only over weekends but denied smoking and substance abuse. On examination, he was conscious and well-oriented, with stable vital signs. Full blood count, liver and renal function tests were within reference ranges, although serum creatinine rose from 48 µmol/L (day 1) to 82 µmol/L on day 2 (Table 1). Serum level of DTG could not be done. Electrocardiography was normal, including a normal QTc. He was managed with intravenous fluid, metoclopramide and an antacid, and was off of his ARVs during his 3-day hospital stay. He was restarted on DTG and TDF/FTC after discharge, day 5 post-overdose. As per hospital protocol, he also had a psychological assessment and psychiatric consultation, which were unremarkable. CD4+ count on day 9 was 538 cells/µL. By day 28 follow-up he reported no serious adverse events and clinical assessment revealed no significant findings. Serum creatinine had also decreased to 72 µmol/L. As at the last check-up 7 months after overdose, the CD4+ count had risen further to 828 cells/µL.

**TABLE 1:** Changes in full blood count and serum chemistry during admission and follow-up.

Complete blood count	Day 1	Day 2	Day 28
**Haemoglobin (g/dL)**	13.7	13.9	14.7
Haematocrit (%)	38.7	41.0	42.2
MCV (fL)	92.1	96.7	93.4
MCH (g/dL)	32.6	32.8	32.5
RDW (%)	12.4	13.2	12.7
**White blood cells ( ë 10^9^/L)**	6.55	4.60	7.61
Neutrophils (%)	44.5	37.7	59.7
Lymphocyte (%)	48.9	53.8	31.3
Monocyte (%)	5.2	4.8	5.1
Eosinophils (%)	1.2	3.3	3.5
Basophil (%)	0.2	0.4	0.3
Platelets (ë10^9^/L)	176	212	220
**Serum chemistry**			
Sodium (mmol/L)	134	138	137
Potassium (mmol/L)	4.2	5.1	4.2
Chloride (mmol/L)	99.8	100.7	100.6
Urea (mmol/L)	4.3	4.0	6.9
Creatinine (µmol/L)	48	82	74
Estimated creatinine clearance (mL/min)	155	91	100
AST (U/L)	31	30	30
ALT (U/L)	28	27	17

MCV, mean corpuscular volume; MCH, mean corpuscular haemoglobin; RDW, red cell distribution width; AST, aspartate aminotransferase; ALT, alanine aminotransferase.

## Discussion

Integrase inhibitors are an essential addition to HIV treatment and act by blocking the incorporation of HIV DNA into the host genome, thus preventing replication. Drugs in this class include raltegravir, elvitegravir and DTG. Dolutegravir is taken once daily (50 mg for adults), has a good tolerability profile and superior virologic activity. It is primarily metabolised by the UDP glucuronosyltransferase (UGT1A1) pathway and excreted into bile. Hepatic cytochrome P4503A also contributes to DTG metabolism through oxidation by a minor pathway.^5^

Dolutegravir can thus be safely used in patients with significant renal dysfunction, without an expectation of renal impairment or dangerously high drug levels. Even though DTG is not known to have any direct effect on glomerular filtration rate, it impairs renal creatinine secretion through inhibition of the organic cation transporter-2 (OCT2) and may create an impression of deteriorating renal function.^5,6^ In addition, Guttierrez et al. mentioned in their review that there was a slight but predictable increase in serum creatinine concentration and a decrease in estimated creatinine clearance (eCrCl) of about 15% among patients taking DTG at the pharmacologic dose and without an actual reduction in glomerular filtration rate.^6^ Our patient experienced a non-progressive but significant increase in baseline serum creatinine, which may be attributable to this effect. Hence, clinicians should be guided by other measures of renal function that are not known to be affected by DTG.

The occurrence of psychiatric symptoms including suicidal ideations in HIV-positive patients receiving DTG was reviewed across five randomised clinical trials by Fettiplace et al., and the authors concluded that similar to other ARV drugs, these symptoms were infrequently reported.^7^ Another review suggested that psychiatric symptoms may indeed be an InSTI class effect. Literature reports of DTG overdose are sparse, and thus its likely effects remain unclear. Lee M et al. reported a case of a suicidal overdose of a combination of DTG, TDF and FTC.^8^ In that case, the authors observed a non-progressive renal failure which was attributed to the effect of DTG, although TDF (a potentially nephrotoxic drug which the patient also used) may have contributed to the rise in serum creatinine. In our patient, where DTG alone was used, there was also a similar non-progressive elevation of serum creatinine. OCT2 inhibition by DTG can readily explain these effects in both cases, and this ‘renal impairment’ is typically reversible without any permanent harm.^8^

To our knowledge, there are no independent research publications regarding single overdose of DTG either in humans or animals. An FDA pre-approval review of non-clinical animal toxicity studies by the drug sponsor concluded that there were no significant adverse events after single-dose ingestion of 10 mg – 1500 mg among rats, mice and monkeys.^9^ However, animals developed gastrointestinal inflammation and haemorrhage in similar studies following repeated short-term, subchronic and chronic daily dosing, at 10–1500 mg/kg/day. In addition, hepatotoxicity was noted in male monkeys, and this included hepatocellular necrosis and diffuse hepatocellular hypertrophy and vacuolation.^9^ These studies also established that the plasma levels achieved by these animals after a maximum of 1500 mg/kg/day are approximately seven times what humans achieve after a 50 mg once daily dose. If the pharmacokinetic relationship between the oral dose and plasma DTG levels remain linear at such doses, it will imply that humans can be expected to experience similar effects after just about 350 mg once daily.

Therapeutic interventions are mostly supportive. Patients are unlikely to benefit from dialysis following an overdose because DTG is highly bound to plasma proteins.^3^ In this index case, we found no significant adverse effect after an overdose of 1500 mg. His minor symptoms disappeared with supportive treatment.

In conclusion, single-ingestion DTG overdose may be associated with minimal toxicity, as compared to repeated daily overdose, which may potentially result in gastrointestinal bleeding and hepatotoxicity. The apparent renal dysfunction noted in these cases is usually owing to OCT2 inhibition by DTG and is reversible. However, as experience is limited to the likely range of toxicities that may develop, physicians are advised to adopt measures that will prevent any form of overdosing using ARVs.
